# Chronic Q Fever with Vascular Involvement: Progressive Abdominal Pain in a Patient with Aortic Aneurysm Repair in the United States

**DOI:** 10.1155/2019/5369707

**Published:** 2019-02-19

**Authors:** Zanthia Wiley, Sujan Reddy, Kara M. Jacobs Slifka, David C. Brandon, John Jernigan, Gilbert J. Kersh, Paige A. Armstrong

**Affiliations:** ^1^Division of Infectious Diseases, Department of Medicine, Emory University School of Medicine, Atlanta, GA, USA; ^2^Division of Healthcare Quality Promotion, Centers for Disease Control and Prevention, Atlanta, GA, USA; ^3^Department of Radiology and Imaging Sciences, Emory University School of Medicine, Atlanta, GA, USA; ^4^Rickettsial Zoonoses Branch, Division of Vector-Borne Diseases, Centers for Disease Control and Prevention, Atlanta, GA, USA

## Abstract

Q fever is a zoonotic bacterial infection caused by *Coxiella burnetii*. Chronic Q fever comprises less than five percent of all Q fever cases and, of those, endocarditis is the most common presentation (up to 78% of cases), followed by vascular involvement. Risk factors for chronic Q fever with vascular involvement include previous vascular surgery, preexisting valvular defects, aneurysms, and vascular prostheses. The most common symptoms of chronic Q fever with vascular involvement are nonspecific, including weight loss, fatigue, and abdominal pain. Criteria for diagnosis of chronic Q fever include clinical evidence of infection and laboratory criteria (antibody detection, detection of *Coxiella burnetii* DNA, or growth in culture). Treatment of chronic Q fever with vascular involvement includes a prolonged course of doxycycline and hydroxychloroquine (≥18 months) as well as early surgical intervention, which has been shown to improve survival. Mortality is high in untreated chronic Q fever. We report a case of chronic Q fever with vascular involvement in a 77-year-old man with prior infrarenal aortic aneurysm repair, who lived near a livestock farm in the southeastern United States.

## 1. Introduction

Chronic Q fever, including endocarditis and vascular involvement, is a rare disease and affects less than 5% of patients with prior acute infection [1]. The mortality rate of acute Q fever is low (less than 2%), but untreated chronic Q fever is generally fatal if untreated [1]. Chronic Q fever with vascular involvement is difficult to diagnose given its rarity and nonspecific symptoms. The most common symptoms include fatigue, abdominal pain, and weight loss [2]. We present a case of Q fever with vascular involvement in a 77-year-old man with prior infrarenal aortic aneurysm repair, who lived near a livestock farm in the southeastern United States.

## 2. Case Report

**Figure 1:**
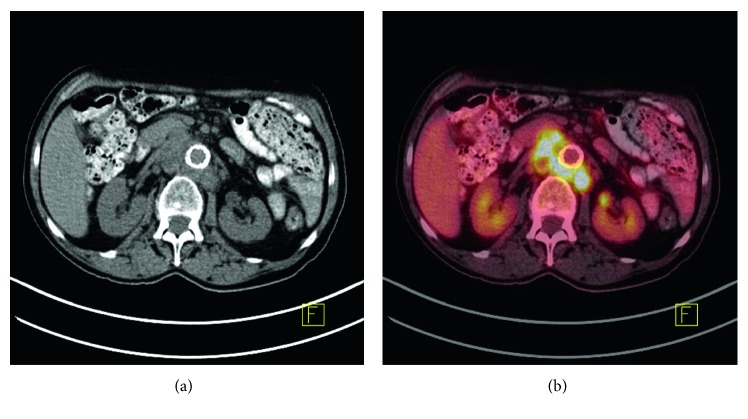
Axial CT and fused FDG PET-CT imaging demonstrating FDG-avid retroperitoneal lymphadenopathy displacing the abdominal aorta anteriorly.

A 77-year-old man with hypertension, hyperlipidemia, and prior infrarenal aortic aneurysm repair presented with several months of worsening lower abdominal pain and a weight loss of 20 pounds (9 kilograms). The endovascular repair of his aortic aneurysm occurred two years prior and was prompted by expansion to 4.4 cm in diameter. He denied any fever or chills. He lived in the rural southeastern United States (Georgia), where he hunted deer and had exposure to livestock on a nearby farm. On exam, he was cachectic and had a temperature of 100.4°F (38°C). Abdominal exam showed tenderness to deep palpation in the epigastrium and bilateral lower quadrants. His white blood cell count was normal at 8.0 × 10^9^/L (reference 4–11 × 10^9^/L), hemoglobin 11.3 g/dL (reference 11.4–14.4 g/dL), and platelet count of 345 × 10^9^/L (reference 150–400 × 10^9^/L). Serum sodium, renal function, and aminotransferases were normal. The erythrocyte sedimentation rate and C-reactive protein were elevated at 100 mm/hr (reference 0–20 mm/hr) and 111.8 mg/dL (reference 0–7.5 mg/dL), respectively. A chest radiograph was unremarkable, and computed tomography (CT) scan of his abdomen and pelvis detected large (up to 2.6 cm × 2 cm) necrotic periaortic lymph nodes with normal appearance of the liver. CT-guided retroperitoneal lymph node biopsies were performed, and pathology was negative for malignancy but noted chronic inflammation and non-necrotizing granulomas. Lymph node aerobic and anaerobic Gram stain and cultures, acid fast bacillus (AFB) smear and culture, and fungal stain and cultures were negative. Aerobic and anaerobic blood cultures, AFB blood smear and culture, serum cryptococcal antigen, HIV antigen/antibody, purified protein derivative for tuberculosis exposure, and rapid plasma reagin (RPR) for syphilis were all negative. Positron emission tomography (PET) CT imaging was pursued for further evaluation of the intra-abdominal lymphadenopathy and revealed increased radiotracer activity and soft tissue stranding at the bifurcation of the aortoiliac stent, indicative of increased metabolic activity (Figure 1).

**Figure 2:**
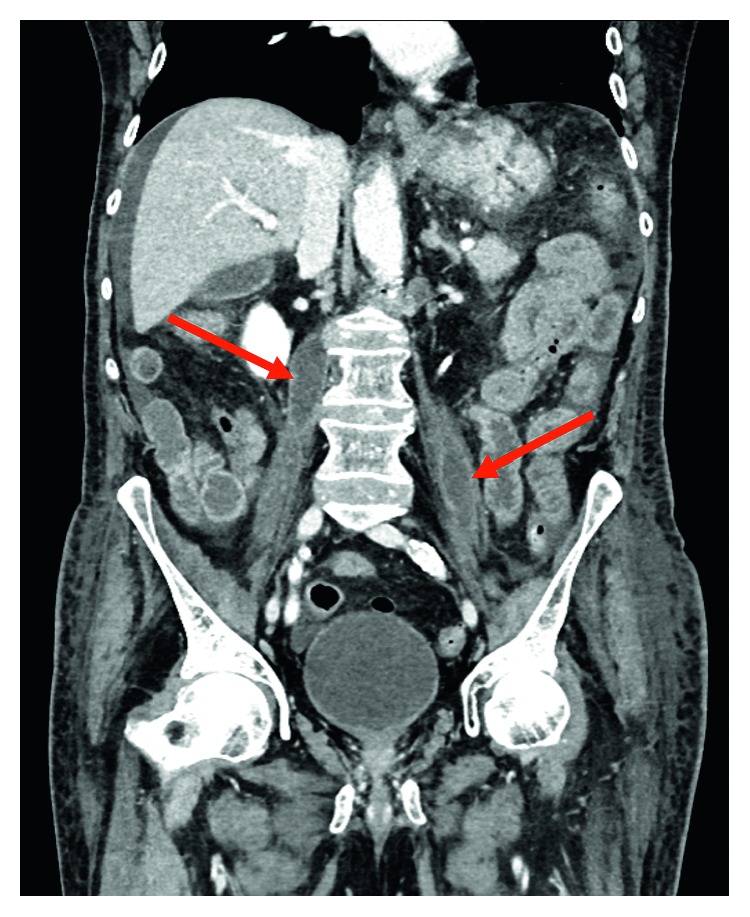
Coronal IV contrast-enhanced CT demonstrating bilateral psoas abscesses (red arrows).

**Figure 3:**
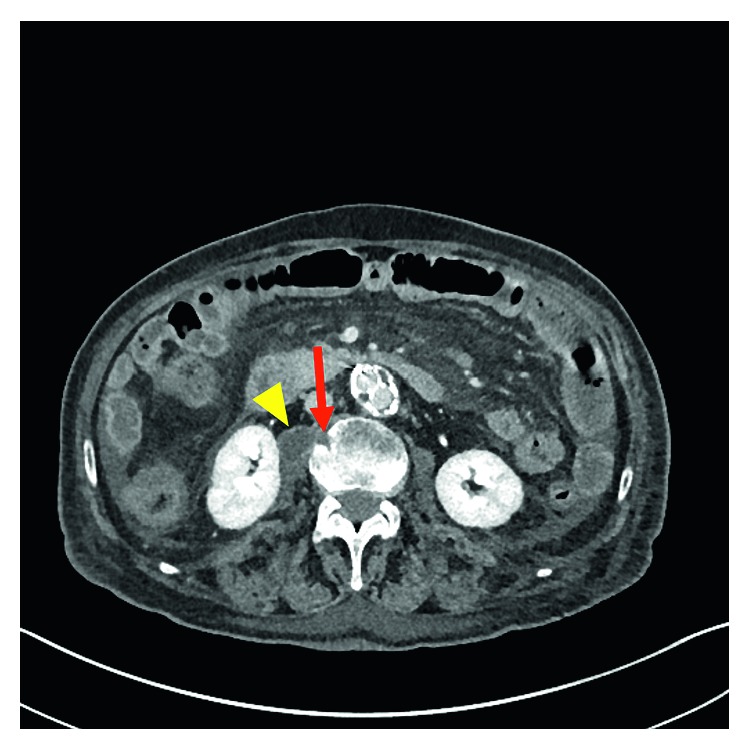
Axial IV contrast-enhanced CT with a small erosion of L2 (red arrow) consistent with osteomyelitis, adjacent to the right psoas abscess (yellow arrowhead).

Prior to hospital discharge, the patient's *Coxiella burnetii* titers were pending as well as the psoas fluid aspirate polymerase chain reaction (PCR) and culture. The psoas fluid aspirate was sent to the Centers for Disease Control and Prevention given negative infectious workup until this point. After discharge, his *Coxiella burnetii* (Q fever) titers resulted as follows: IgM Phase I: 1 : 64, IgG Phase I: 1 : 131,072, IgM Phase II: 1 : 16, IgG Phase II: 1 : 65,536. Polymerase chain reaction (PCR) testing of the psoas fluid aspirate was pursued, and aspirate PCR and cultures were positive for *Coxiella burnetii*. During outpatient infectious diseases management, the patient was started on therapy with hydroxychloroquine and doxycycline. Despite therapy, he had progressive decline in his functional and nutritional status, which limited monitoring of titers and drug levels. On a subsequent admission two months later, he was noted to have developed psoas abscesses bilaterally (left 1.9 × 1.8 × 4 cm and right 1.9 × 2.5 × 6 cm) (Figure 2) with aortitis of the posterior aspect of the aorta at the level of L2 and osteomyelitis of L2 and L3 vertebral bodies (Figure 3). The patient declined despite therapy with doxycycline and hydroxychloroquine and fluoroscopy-guided drainage (with drain placement) of the psoas abscesses. The patient was deemed high-risk given need for revision of aneurysm repair, progression to bedbound status, failure to thrive, and poor nutritional status. After discussions with the patient, family, and the medical and surgical teams, it was decided to forego potential surgical interventions, such as removal of the infected graft, due to the high risk of intraoperative mortality. The patient and his family elected hospice care. He died at home five months after the diagnosis of chronic Q fever and four years after his abdominal pain began. The exact timing of his exposure to *C. burnetii* is uncertain, but he did recall that several years prior to the onset of symptoms, he assisted his neighbor with the difficult birth of a calf.

## 3. Discussion

Q fever is caused by infection with the bacteria *Coxiella burnetii*. Most people are exposed when they inhale dust contaminated by urine, feces, milk, or birth products of infected animals [1]. Infected birth products can contain particularly high bacterial loads. *Coxiella burnetii* is highly virulent, and inhalation of even one organism can cause infection, yet many chronic Q fever patients do not recall the precise exposure, or even the episode of acute illness. Acute Q fever is defined as fever in addition to one or more of the following: rigors, retrobulbar headache, acute hepatitis, pneumonia, or elevated liver enzyme levels [1]. Asymptomatic infections may also occur [1]. Acute Q fever is laboratory confirmed by the presence of one of the following: fourfold change in serology IgG-specific antibody titer to *C. burnetii* Phase II antigen by direct immunofluorescence (IFA) between paired samples, or detection of *C. burnetii* DNA in a clinical specimen via amplification of a specific target by polymerase chain reaction (PCR) assay, or demonstration of *C. burnetii* in a clinical specimen by immunohistochemical methods (IHC), or isolation of *C. burnetii* from a clinical specimen by culture. A laboratory supportive case of acute Q fever includes a single supportive IFA IgG titer of ≥1 : 128 to Phase II antigen or serologic evidence of elevated Phase II IgG or immunoglobulin M (IgM) antibody reactive with *C. burnetii* antigen by enzyme-linked immunosorbent assay (ELISA), dot-ELISA, or latex agglutination [1].

Chronic Q fever comprises less than five percent of all Q fever cases and, of those, endocarditis is the most common presentation (60–78% of cases), followed by vascular involvement [1, 3, 4]. Clinical criteria include newly diagnosed, culture-negative endocarditis, particularly in patients with known valvulopathy or immunocompromised state [1]. Clinical criteria also include suspected vascular infection in patients with known vascular aneurysm or vascular prosthesis, or osteomyelitis, osteoarthritis, chronic hepatitis, or pneumonitis in the absence of other known etiology [1]. Chronic Q fever is laboratory confirmed by one of the following: serologic evidence of IgG antibody to *C. burnetii* Phase I antigen ≥1 : 800 by IFA, detection of *C. burnetii* DNA in a clinical specimen via amplification of a specific target by PCR assay, demonstration of *C. burnetii* antigen in a clinical specimen by IHC, or isolation of *C. burnetii* from a clinical specimen by culture [1]. Laboratory supportive data include an antibody titer to *C. burnetii* Phase I IgG antigen ≥1 : 128 and <1 : 800 by IFA [1].

Major risk factors for developing chronic Q fever with vascular involvement include previous vascular surgery, preexisting valvular defects, aneurysms, vascular prosthesis, age, and renal insufficiency [5]. The diagnosis can be difficult given the rarity of the disease as well as the nonspecific nature of the presenting symptoms. In a Dutch national database cohort study of 122 patients with vascular chronic Q fever, the most common presenting symptoms reported were nonspecific and included fatigue, abdominal pain, and weight loss. Only 18 (14.8%) of these chronic vascular Q fever patients had fever. In this group, 62 (50.8%) had infection at the site of a vascular graft. Overall mortality was 23.7% (29), and the most common cause of death was ruptured aneurysm [2].

The treatment of chronic Q fever includes doxycycline and hydroxychloroquine (100 mg of doxycycline twice daily with 200 mg of hydroxychloroquine three times daily) for 18–24 months [1]. Some patients do not respond to antibiotic therapy; in these cases, early surgical intervention may improve patient survival [1, 6, 7]. A study of 100 patients with Q fever vascular infection found that surgery was the only factor that lowered overall mortality at 2.5 years (11.2% vs 27.6% of deaths, *P*=0.19). Surgery was also associated with decreasing serology titers (74.1 vs 57.1%, *P*=0.03) [8].

Chronic Q fever with vascular involvement is a rare disease with high mortality. The diagnosis requires clinical consideration and investigation of patient history including potential animal exposures, thorough diagnostic workup (serology, culture, PCR, and imaging), and a multidisciplinary approach to treatment. Inquiries about potential animal exposures are important, but explicit exposure is often absent. Since *Coxiella burnetii* transmission often occurs via the air (up to kilometers from infected animal), the diagnosis of chronic Q fever with vascular involvement should be considered in any case of aortitis/mycotic aneurysm and periaortic abscess of unknown etiology [9–11].
